# Accuracy of the spontaneous breathing trial using a combined CPAP + PSV model to predict extubation outcomes in very preterm infants

**DOI:** 10.1186/s12887-022-03642-2

**Published:** 2022-10-31

**Authors:** Zhe Li, Jiang Xue, Xin-Yuan Guo, Fang Wang, Xue Zhang, Qi Li, Jing-Liang Tang, Juan Ji, Guang-Jun Du

**Affiliations:** 1 Department of Anesthesiology, Shandong Provincial Hospital Affiliated to Shandong First Medical University, 250021 Jinan, Shandong China; 2 Department of Neonatology, The Second Hospital, Cheeloo College of Medicine, Shandong University, 250033 Jinan, Shandong China; 3 Department of Rheumatology, Qilu Hospital, Cheeloo College of Medicine, Shandong University, 250012 Jinan, Shandong China; 4 School of Ethnic Medicine, Key Laboratory of Chemistry in Ethnic Medicinal Resources, State Ethnic Affairs Commission & Ministry of Education, Yunnan Minzu University, 650500 Kunming, Yunnan, China; 5 Department of Neonatology, Feixian People’s Hospital, 273400 Feixian, Shandong China

## Abstract

**Background:**

Very preterm infants often require mechanical ventilation. However, objective criteria to predict the outcomes of extubation in very premature neonates remain lacking. The aim of this study was to investigate the accuracy of the spontaneous breathing trial (SBT) using a combined model of continuous positive airway pressure (CPAP) and low-level pressure support ventilation (PSV) to predict the extubation outcomes of preterm infants with gestational age < 32 weeks.

**Methods:**

Preterm infants with gestational age < 32 weeks, birth weight < 1500 g and requiring mechanical ventilation were selected for the study. All infants underwent a 10-minute SBT using CPAP combined with low-level PSV prior to the planned extubation. Then, the infants were extubated within 1 h after SBT. The outcomes of extubation were considered successful if the neonates did not require reintubation 72 h after extubation.

**Results:**

A total of 119 eligible preterm infants were enrolled in the study, with a median gestational age of 28.9 (27.1–30.3) weeks and a median birth weight of 1100 (900–1350) g. In total, 101 of all infants had successful extubation, 18 of whom failed and eventually had to be reintubated. Of the 102 infants who achieved SBT, 99 were successfully extubated, and 15 of the 17 infants who did not pass SBT had failed extubation. Finally, the diagnostic value for SBT could be assessed with a sensitivity of 98%, a specificity of 83.3%, a positive predictive value of 97.1% and a negative predictive value of 88.2%.

**Conclusion:**

SBT using a combined CPAP + low-level PSV model can predict the outcomes of extubation in very preterm infants with high sensitivity and specificity.

## Introduction

Invasive mechanical ventilation is a common life support technique for very preterm infants (gestational age < 32 weeks) [1], but prolonged mechanical ventilation can lead to lung injury and an increased risk of complications, such as ventilator-associated pneumonia and bronchopulmonary dysplasia [1–3]. To reduce the occurrence of related complications, clinicians try to have objective criteria to determine the timing of extubation, aiming for earlier extubation and a shorter duration of invasive mechanical ventilation [4]. Currently, uniform criteria for extubation in very preterm infants are lacking, and the decision to extubate an infant is usually a subjective clinical judgement made by the attending physician based on the infant’s ventilator parameters, arterial blood gas values and overall clinical stability. However, incorrect clinical judgement often leads to failed extubation [5]. Studies have shown that infants with failed extubation face respiratory setback [6] and are at increased risk of bronchopulmonary dysplasia, distant neurodevelopmental impairment and a prolonged need for respiratory support [7–9]. Therefore, very preterm infants need to be extubated at the appropriate time to avoid the adverse effects of prolonged intubation and the potential risks associated with premature extubation failure. At present, we still lack objective criteria to determine the optimal timing of extubation in very preterm infants.

In recent years, the spontaneous breathing trial (SBT) has been increasingly used in the assessment of neonates before extubation [5, 10–12]. SBT is a tool to assess the ability of infants requiring mechanical ventilation to breathe spontaneously and refers to receiving minimal or no ventilatory support while the infant is still intubated. This approach allows the infant to breathe spontaneously, during which time the outcome of the trial is determined by the occurrence of various clinical events, such as apnoea, bradycardia and decreased oxygen saturation [11]. Several studies [11–13] have investigated the accuracy of SBT in predicting successful extubation in preterm infants, suggesting that SBT has a high sensitivity but moderate specificity. However, a multicentre study [14] refuted the value of SBT in the assessment of extubation in extremely preterm infants. In these studies [11–14]_,_ the mode of ventilatory support and the duration of the trial used to perform the spontaneous breathing trial varied, which may have led to differences in their results. Currently, uniform definitions of the mode of ventilatory support and the duration of the trial when performing a spontaneous breathing trial in preterm infants are lacking. In previous studies [11, 13–15], SBT has mostly been performed using 3–5 min of endotracheal continuous positive airway pressure (ET-CPAP) with PEEP to 4–6 cmH2O, whereas in this study, we selected very preterm infants with gestational age < 32 weeks as the study population, and all infants were given 10 min of endotracheal continuous positive airway pressure combined with low-level pressure support ventilation (CPAP + PSV) prior to extubation. The aim of this study was to assess the accuracy of the combined CPAP + PSV mode of SBT in predicting the outcome of extubation in very preterm infants.

## Objects & methods

### Objects

This work is a single-centre prospective study, and very preterm infants admitted to the Neonatal Intensive Care Unit (NICU) of the Affiliated Hospital of Shandong University between January 1, 2020 and March 31, 2022 who required mechanical ventilation and whose gestational age was < 32 weeks and birth weight was < 1500 g, were selected for the study. Inclusion criteria: ① gestational age < 32 weeks, birth weight < 1500 g; ② mechanical ventilation with tracheal intubation for more than 12 h; and ③ first intubation after birth never removed. Exclusion criteria: ① congenital inherited metabolic diseases and severe congenital malformations; ② self-extubation (excluding cases of accidental extubation followed by immediate intubation and continued mechanical ventilation); ③ diseases that interfere with extubation, such as central hypoventilation syndrome and severe perinatal asphyxia; and ④ other reasons (e.g., surgery) requiring continued intubation.

All parents of the infants gave informed consent to participate in the study and signed an informed consent form when the infants met the inclusion criteria. This study was approved by the Research Ethics Committee of the Second Affiliated Hospital of Shandong University.

### Clinical data collection

The following clinical data were collected on the infants: gestational age, birth weight, sex, maternal mode of delivery, antenatal steroids, use of pulmonary surfactant, 1-minute and 5-minute Apgar scores, medications used by infants (e.g., caffeine, steroids, or inotropes), duration of mechanical ventilation, respiratory support parameters before extubation, and postmenstrual gestational age at extubation.

### Preparation phase for extubation

The following basic conditions [16] for extubation need to be met before an infant can be extubated: (1) stable bedside clinical parameters, i.e., heart rate stable within normal range, no bradycardias, good respiratory drive with respiratory rate above backup rate, O2 saturations stable within target limits 91–95%; (2) low ventilator parameters, i.e., stable oxygenation at FiO2 ≤ 0.4, MAP consistently ≤ 7–9 cmH2O, backup rate below the spontaneous respiratory rate, typically 20–30; (3) acceptable blood gas parameters, i.e., pH ≥ 7.20 and PaCO2 < 60 mmHg. Once these basic conditions have been met, the attending physician will plan for the baby’s extubation and decide on the exact timing of the extubation based on the infant’s circumstances.

### Spontaneous breathing trial (SBT)

When the attending physician clinically judged that the infant included in the study had met the criteria for extubation, she left the infant’s bedside before preparing for extubation and informed the experimenters to perform a 10-minute SBT on the infant with the SLE 5000 ventilator. The experimenter adjusted the ventilator to PSV mode and PEEP to 5 cmH2O, PS to 10 cmH2O, respiratory rate to 1 and the duration of the trial to 10 min. During this 10-minute trial, the experimenter was responsible for recording the infant’s heart rate and oxygen saturation and for observing the occurrence of abnormalities, such as cyanosis, apnoea and respiratory distress. If no bradycardia, oxygen desaturation or apnoea occurred during the trial, the trial was judged to be successful, and the infant remained mechanically ventilated at the end of the 10-minute SBT with the original respiratory support parameters. If clinical signs of apnoea, decreased oxygen saturation or bradycardia occur during this time, which indicated that the trial had failed, the ventilator was immediately adjusted for the original respiratory support treatment without waiting 10 min for the trial to end. All infants, irrespective of the success of the SBT, were extubated by the attending physician within 1 h of SBT (the attending physician responsible for extubation was not present during the infant’s SBT and was unaware of the SBT results). After extubation, the attending physician administered reasonable noninvasive ventilation based on the infant’s respiratory status (the experimenter in charge of the SBT was not involved in the decision to extubate the infant and the choice of postextubation respiratory support). If the infant required reintubation within 72 h of extubation, the infant was considered to have failed extubation [11]_,_ and the reasons for reintubation, the time to reintubation and the possible complications (e.g., sepsis, necrotizing enterocolitis, etc.) for the infant who failed extubation were analysed and recorded. If the infant remained extubated for more than 72 h without reintubation, extubation was considered successful.

SBT failure was defined when one of the following situations occurred despite the FiO2 being increased by up to 15%: ① bradycardia (heart rate < 100 beats/min) for more than 15 s; ② a decrease in percutaneous oxygen saturation (SpO2 < 85%) for more than 15 s; ③ apnoea ≥ 1 that could not be recovered after stimulation.

### Indications for reintubation

The following conditions occurred in infants with noninvasive ventilator-assisted ventilation: ① Apnoea ≥ 3 times in 1 h that requires stimulation to recover; ② respiratory acidosis (PaCO2 > 65 mmHg and pH < 7.20); and ③ increased oxygen demand, i.e., requiring FiO2 > 60% to maintain SpO2 in the target range (91-95%).

### Statistical analysis

The SPSS 26.0 statistical software was used for data analysis. Normally distributed measures are expressed as the mean ± standard deviation. Nonnormally distributed measures are expressed as the median M (interquartile range, IQR), and the rank sum test was used for comparisons between groups. Count data are expressed as the number of cases (percentage), and the chi-square test was used for comparisons between groups. Univariate and multivariate logistic regression analyses were used in an exploratory attempt to investigate the association between all relevant clinical factors and success of extubation. Differences were considered statistically significant at P < 0.05. The sensitivity, specificity, positive predictive value (PPV) and negative predictive value (NPV) of SBT were calculated using the extubation outcome (success/failure) as the reference standard.

## Results

**Fig. 1:**
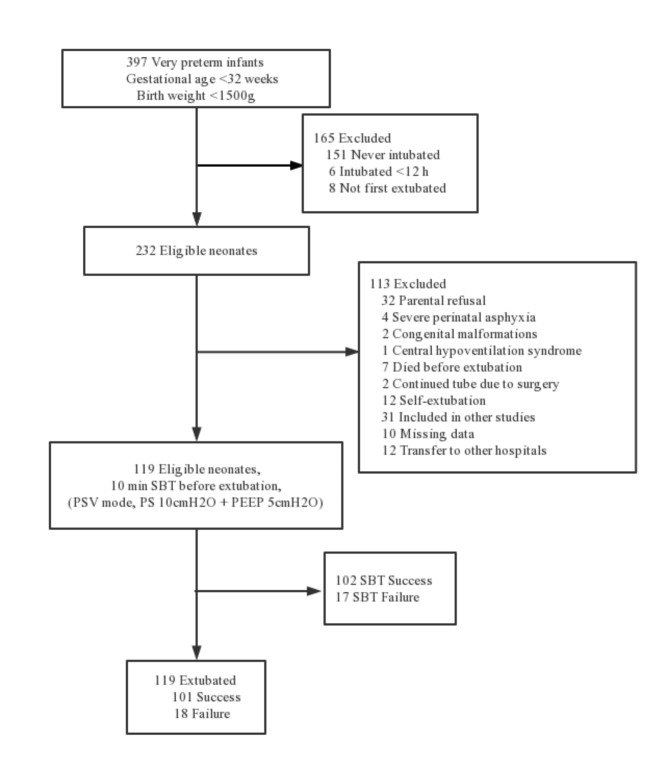
Selection criteria for enrolling infants and the process of the spontaneous breathing trial

A total of 397 infants with gestational age < 32 weeks were treated in the NICU of the Affiliated Hospital of Shandong University during the study period, of which 232 were eligible for the study and 113 were subsequently excluded according to the exclusion criteria, resulting in a total of 119 infants being included in the study and undergoing a 10-minute spontaneous breathing trial prior to extubation, as shown in Fig. 1.

Of the 119 infants included in this study, 77 were male and 42 were female, with a median gestational age of 28.9 (27.1–30.3) weeks and a median birth weight of 1100 (900–1350) g. Finally, 101 infants were successfully extubated, and 18 infants failed. The general clinical characteristics of infants with successful and failed extubation are shown in Table 1.

**Table 1:** General clinical characteristics of infants with successful and failed extubation

Clinical variable		Success group (n = 101)	Failure group (n = 18)	P-value
Gestational age/weeks		29.0(27.3 ~ 30.5)	27.6(26.2 ~ 29.4)	0.044^a^
Birth weight (g)		1135(900 ~ 1380)	1010(817 ~ 1215)	0.079^a^
Gender (M/F)		65/36	12/6	0.850^b^
Mode of delivery (VD/CS)		27/74	6/12	0.771^b^
Antenatal steroids (Yes/No)		78/23	13/5	0.873^b^
Apgar score(at 1 min)		8(6 ~ 10)	7(5 ~ 9)	0.225^a^
Apgar score(at 5 min)		9(8 ~ 10)	9(8 ~ 10)	0.205^a^
Pulmonary surfactant (Yes/No)		54/47	5/13	0.037^b^
Steroids used for infants (Yes/No)		6/95	4/14	0.067^b^
Caffeine use (Yes/No)		82/19	15/3	1^b^
Inotropes use (Yes/No)		9/92	2/16	1^b^
Postmenstrual gestational age at extubation/weeks		29.7(28.2 ~ 30.9)	29.5(28.2 ~ 30.7)	0.689^a^
PEEP before extubation /cmH2O		5(5 ~ 6)	6(5 ~ 6)	0.017^a^
PIP before extubation /cmH2O		15.5(15 ~ 17)	18(17 ~ 18.5)	0.001^a^
FiO2 before extubation /%		24.5(21 ~ 27)	26(25 ~ 35)	0.004^a^
Duration of mechanical ventilation /d		3(1 ~ 5)	6.5(2.8 ~ 19.8)	0.010^a^
Post-extubation support^c^	Room air	17	1	-
	NIPPV	32	11	-
	nCPAP	23	4	-
	BiPAP	19	2	-
	O2	10	0	-

M: male, F: female, VD: vaginal delivery, CS: Cesarean section

a: rank sum test, b: chi-square test

c: unable to run Chi-Square test because of the small sample size

A multivariate logistic regression model with all relevant clinical factors was performed and adjusted with the stepwise regression method, as shown in Table 2. The duration of ventilation had been included in the original multivariate logistic regression analysis (OR = 0.971, 95%CI: 0.896 ~ 1.052, P = 0.469), and then, was excluded by the adjusted multivariate analysis with stepwise regression method. The adjusted multivariate analysis revealed that higher gestational age and use of pulmonary surfactant had a significant association with success of extubation (odds ratio = 2.1, 95% CI: 1.4 ~ 3.2, P = 0.001, odds ratio = 41.7, 95% CI: 5.2 ~ 331.9, P < 0.001, respectively). The wide confidence interval was possibly due to our limited sample size.

**Table 2:** Adjusted multivariate analysis with stepwise regression method for successful extubation

Clinical variable	Odds ratio	95% CI	*P*
Gestational age	2.1	1.4 ~ 3.2	0.001
Surfactant use	41.7	5.2 ~ 331.9	< 0.001
PIP prior to extubation	0.8	0.6 ~ 1.0	0.08
FiO2 prior to extubation	0.9	0.7 ~ 1.0	0.044

Note: Adjusted for gestational age, surfactant use, PIP prior to extubation, FiO2 prior to extubation

All 119 infants underwent a spontaneous breathing trial prior to extubation, with 102 infants with successful SBT and 17 infants with failed SBT. Ultimately, we obtained the results of the validated indicators of SBT for predicting extubation outcome in very preterm infants, as shown in Table 3. The main reasons and time points for SBT failure in the 17 infants are shown in Table 4.

**Table 3:** Validated indicators of SBT for predicting the outcome of extubation

SBT results	Extubation		
	Success	Failure	Total
Success	99	3	102
Failure	2	15	17
Total	101	18	119

Sensitivity = 98%

Specificity = 83.3%

Positive Predictive Value (PPV) = 97.1%

Negative Predictive Value (NPV) = 88.2%

**Table 4:** Reasons and time points for SBT failure

Projects	Reasons for SBT failure				Time points for SBT failure		
	O2 saturation decreases	Bradycardia	Apnoea	Total	Failure in 1–5 min	Failure in 6–10 min	Total
Number	11	4	2	17	14	3	17

After the infants were extubated, the attending physicians selectively administered reasonable noninvasive respiratory support treatment according to the infant’s respiratory condition. The respiratory support modalities given to all 119 infants after extubation are shown in Table 1; we were unable to run chi-square tests because of the small sample size.

The average time to reintubation for the 18 infants with failed extubation was 34.67 ± 18.62 h. The reasons for reintubation and the complications associated with reintubation are shown in Table 5.

**Table 5:** Reasons and related complications for reintubation

Projects	Reasons for reintubation					Complications associated with reintubation				
	Mixed factors	Apneas	Respiratory acidosis	Increased O2 demand	Total	Pneumothorax	NEC	PPHN	None	Total
Number	4	5	5	4	18	1	1	1	15	18

PPHN: persistent pulmonary hypertension of the newborn;NEC: necrotizing enterocolitis

## Discussion

This work is a single-centre prospective study that objectively evaluated the value of the combined CPAP + PSV mode of spontaneous breathing trial for clinical use in guiding the extubation of very preterm infants. All very preterm infants included in the study underwent a 10-minute spontaneous breathing trial prior to extubation, and the infants’ SBT results and extubation outcomes were observed and recorded. We found that the combined CPAP + PSV model of SBT could predict extubation outcome in very preterm infants at gestational age < 32 weeks with a sensitivity of 98%, specificity of 83.3%, positive predictive value of 97.1% and negative predictive value of 88.2%. The value of SBT in predicting successful extubation in preterm infants has also been reported in previous relevant studies [11–13], suggesting a high sensitivity (92-97%) but only moderate specificity (50-81.4%) of SBT. In comparison, the SBT used in this study had higher sensitivity and specificity in predicting extubation outcome in very preterm infants, which may be related to the difference in gestational age and birth weight of the preterm infants selected in this study, as well as the more reasonable mode of ventilatory support and duration of the trial used for the SBT in this study.

Fiatt et al. [17] found that 62.3% of neonates required NIPPV after extubation, whereas 36.1% of infants in our sample required NIPPV after extubation and 17.7% required BiPAP after extubation. For infants requiring NIPPV and BiPAP after extubation, CPAP alone is not sufficient to maintain normal oxygenation, and the administration of CPAP alone within the tracheal tube may therefore also lead to SBT failure in this group of successfully extubated infants. An ET-CPAP model of SBT was used in the study by Shalish et al. [14], yielding a low specificity and negating the value of SBT. In this study, we selected a combined CPAP + PSV mode of ventilatory support at the time of SBT to reduce the likelihood of SBT failure in some infants who required NIPPV and BiPAP after successful extubation. Furthermore, we used low levels of PSV during SBT, thus reducing the likelihood that some infants could have a successful SBT with high-pressure support ventilation but ultimately face failed extubation. In this study, we used a combined mode of SBT with endotracheal CPAP and low levels of PSV, which resulted in a higher sensitivity and specificity.

In addition, most previous relevant studies of SBT have used a trial duration of 3–5 min [11, 13–15], whereas this study examined a trial duration of 10 min, which could identify more infants who faced SBT failure after 5 min. A study by Eissa et al. [12] also chose a 10-minute SBT duration, and their study found that SBT failure occurred in 118 of 465 infants with gestational age ≤ 30 weeks, of which 91 (77.1%) SBT failures occurred at 3–5 min of the trial, while 27 (22.9%) occurred at 6–10 min. In this study, a total of 17 infants failed SBT, of which 3 (17.6%) were judged to have failed at 6–10 min of the trial. These 3 infants eventually failed extubation; therefore, the 10-minute trial duration avoids missing infants who fail SBT after 5 min and ultimately identifies more infants who fail extubation, which increases the specificity of SBT. Chawla et al. [11] also studied very preterm infants with gestational age < 32 weeks, but they performed SBT with a 5-minute ET-CPAP modality, yielding a sensitivity of 92% and specificity of 50% for SBT. These values are both lower than those obtained in this study, which suggests that the 10-minute combined ET-CPAP + PSV model of SBT used in this study is more reasonable.

The failure rate of extubation in preterm infants in this study was 15.1% compared to the 20.4-29.2% failure rates in other studies [11–14]. This variability may be due differences in the preterm infants selected among different studies or differences in the time window for observation of extubation failure. The window of observation for failed extubation in currently published studies is inconsistent. A systematic review [18] showed that the time window of observation for failed extubation ranged from 12 h to 7 days after extubation, with commonly used windows including 48 h (29%), 72 h (35.5%) and 168 h (25.8%) after extubation. In this study, we limited the observation window for infant extubation failure to 72 h after extubation to capture most of the clinical information on extubation failure and to avoid including new disease factors, such as late onset sepsis and necrotizing enterocolitis as causes of extubation failure. Similarly, many studies have defined reintubation within 72 h of extubation as extubation failure [17, 19].

When an infant breathes spontaneously under tracheal intubation, the airway resistance is high, and giving the tracheally intubated infant appropriate pressure support can overcome the airway resistance because of the weakness of the respiratory muscles in preterm infants. Related studies have shown [20] that a 5-minute ET-CPAP performed before extubation in extremely preterm infants produces a significant respiratory load. In this study, we chose a combined ET-CPAP + PSV mode of support, which gave pressure support to the infant, thereby reducing respiratory resistance. Furthermore, the trial was immediately terminated as a failure and the original mechanical ventilation support treatment was continued when the infant showed one of the following signs during the spontaneous breathing trial, bradycardia > 15 s, apnoea that did not resume despite stimulation, or a decrease in oxygen saturation > 15 s. Thus, the impact of SBT on the clinical stability of the infant was avoided. However, a recent study [21] has shown that a 30-minute spontaneous breathing trial is safe for preterm infants. Therefore, different SBT durations may be appropriately selected in the future for a stratified analysis to obtain a more appropriate trial duration.

In addition, adjusted factors associated with successful extubation included a greater gestational age, the use of surfactant, and a lower preextubation FiO2. Similar findings were obtained in a study by Gupta et al. [22]. We found that it seems to be able to avoid extubation failure with the reasonable use of surfactant, but this conclusion is needed to be explored in further research. Firstly, the study population selected for this trial was very preterm infants requiring mechanical ventilation, not all of whom had neonatal respiratory distress syndrome (NRDS) and required pulmonary surfactant therapy. Some of these infants were born with a combination of pneumothorax, pulmonary hypertension, sepsis, spontaneous intestinal perforation and other conditions that required mechanical ventilation. They were not eligible for surfactant. There are also some infants with NRDS who are eligible for surfactant but whose parents refuse to use it for personal reasons. We had tried to select only this group of infants who meet the indications for surfactant use without the influence of other diseases, but failed to conduct further analysis due to our limited sample size. Nevertheless, it is necessary for very preterm infants with NRDS to be treated with surfactant in a reasonable and timely manner.

This study was also subject to limitations. First, this work is a single-centre study, and the findings may not be fully applicable to other institutions. Furthermore, the extubation of preterm infants widely varied by gestational age, and the sample size in this study was limited to allow further analysis of the study population stratified by gestational age. In the future, we could conduct a multicentre study and stratify the analysis according to the different gestational ages of the infants to obtain more authoritative results.

## Conclusion

The results of this study show that the combined CPAP + low-level PSV model of SBT can predict the outcome of extubation in very preterm infants at gestational age < 32 weeks with high sensitivity, specificity, PPV and NPV. Therefore, physicians may consider using a combined CPAP and low-level PSV model of SBT to guide extubation of very preterm infants with gestational age < 32 weeks in clinical practice.

## Data Availability

The datasets used and/or analyzed during the current study are available from the corresponding author on reasonable request.

## List of abbreviations

SBT: Spontaneous breathing trial.
CPAP: Continuous positive airway pressure.
PSV: Pressure support ventilation.
PS: Pressure support.
ET-CPAP + PSV: Endotracheal CPAP combined with PSV.
ET-CPAP: Endotracheal continuous positive airway pressure.
NICU: Neonatal Intensive Care Unit.
PIP: Peak inspiratory pressure.
PEEP: Positive end-expiratory pressure.
FiO2: Fraction of inspiration O2.
SpO2: Percutaneous oxygen saturation.
IQR: Interquartile range.
PPV: Positive predictive value.
NPV: Negative predictive value.
NIPPV: Noninvasive intermittent positive pressure ventilation.
BiPAP: Bi-level positive airway pressure.
nCPAP: Nasal continuous positive airway pressure.
PPHN: Persistent pulmonary hypertension of the newborn.
NEC: Necrotizing enterocolitis.
NRDS: Neonatal respiratory distress syndrome.
